# Absence of edge reconstruction for quantum Hall edge channels in graphene devices

**DOI:** 10.1126/sciadv.adf7220

**Published:** 2023-05-12

**Authors:** Alexis Coissard, Adolfo G. Grushin, Cécile Repellin, Louis Veyrat, Kenji Watanabe, Takashi Taniguchi, Frédéric Gay, Hervé Courtois, Hermann Sellier, Benjamin Sacépé

**Affiliations:** ^1^ Université Grenoble Alpes, CNRS, Grenoble INP, Institut Néel, Grenoble 38000, France.; ^2^ Université Grenoble Alpes, CNRS, LPMMC, Grenoble 38000, France.; ^3^ Research Center for Functional Materials, National Institute for Materials Science, 1-1 Namiki, Tsukuba 305-0044, Japan.; ^4^ International Center for Materials Nanoarchitectonics, National Institute for Materials Science, 1-1 Namiki, Tsukuba 305-0044, Japan.

## Abstract

Quantum Hall (QH) edge channels propagating along the periphery of two-dimensional (2D) electron gases under perpendicular magnetic field are a major paradigm in physics. However, groundbreaking experiments that could use them in graphene are hampered by the conjecture that QH edge channels undergo a reconstruction with additional nontopological upstream modes. By performing scanning tunneling spectroscopy up to the edge of a graphene flake on hexagonal boron nitride, we show that QH edge channels are confined to a few magnetic lengths at the crystal edges. This implies that they are ideal 1D chiral channels defined by boundary conditions of vanishing electronic wave functions at the crystal edges, hence free of electrostatic reconstruction. We further evidence a uniform charge carrier density at the edges, incompatible with the existence of upstream modes. This work has profound implications for electron and heat transport experiments in graphene-based systems and other 2D crystalline materials.

## Teaser

Scanning tunneling spectroscopy in graphene reveals ideal quantum Hall edge channels, strongly confined at the physical edge.

## INTRODUCTION

In 1982, 2 years after the discovery of the quantum Hall (QH) effect (*1*), Halperin (*2*) predicted the existence of edge states carrying the electron flow along sample periphery. These edge states, which form unidirectional (chiral) ballistic conduction channels, have been pivotal in understanding most of the transport properties of the QH effect (*3*, *4*). They have served as an extraordinarily versatile platform for a multitude of quantum coherent experiments (*5*), culminating recently in the evidence of fractional statistics in the fractional QH effect (*6*) and the possibility of anyon braiding through interferometry (*7*).

**Fig. 1.**
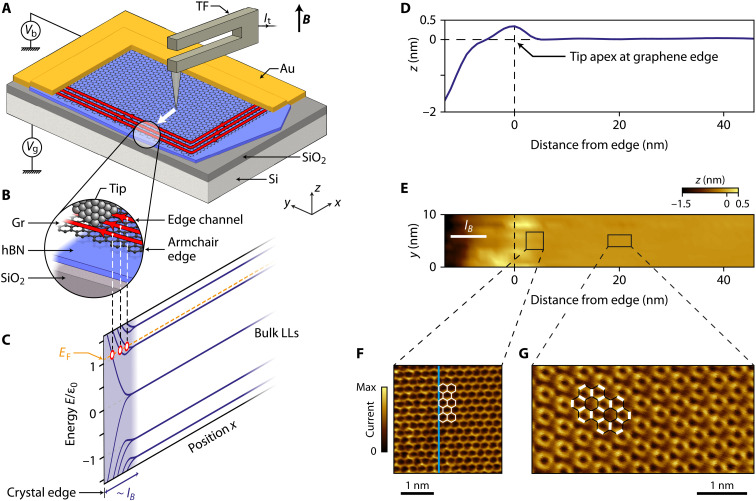
Tunneling spectroscopy of QH edge states. (**A** and **B**) Schematics of the experiment. A PtIr tip is glued at the extremity of one prong of a piezoelectric tuning fork to enable imaging both in scanning tunneling microscopy (STM) (by regulating the tunneling current *I*_t_) and in atomic force microscopy (AFM) (by regulating the frequency shift of the tuning fork). Graphene lies atop an insulating hBN flake and is contacted by a Cr/Pt/Au electrode to apply the sample bias *V*_b_. A back-gate voltage *V*_g_ applied to the Si/SiO_2_ substrate enables to tune the Fermi level *E*_F_ in graphene. Graphene edges are first located by AFM under perpendicular magnetic field, ***B***. The tip is then moved from the graphene bulk to the edge in STM to perform tunneling spectroscopy of QH edge channels. (**C**) Landau level spectrum (*16*–*18*) as a function of energy *E* (normalized to the first cyclotron gap ε_0_) and position. The Landau levels disperse at an armchair edge on the scale of the magnetic length *l_B_*. Their intersect with the Fermi level defines the QH edge channels. (**D** and **E**) Topographic image (E) and its *z* profile averaged on the *y* direction (D) of the graphene edge obtained in STM. We consider that the tip apex is located above the graphene edge at the maximum of the *z* profile. (**F**) Atomic resolution of the graphene honeycomb lattice measured in STM a few nanometers away from the edge. The vertical blue line indicates the crystal edge orientation deduced from (E). (**G**) Kekulé bond order imaged in charge-neutral graphene (*30*) at *V*_g_= −5 V at a distance of 20 nm from the edge.

The existence of edge states was initially inferred as a consequence of the boundary conditions imposed by the physical edges on the electron wave functions (*2*). The energy of the electron states that are condensed into Landau levels increases upon approaching the edge due to the hard-wall boundary conditions, opening conduction channels—the QH edge channels—spatially located at their intersection with the Fermi level (see Fig. 1, B and C) (*2*). Inclusion of a smooth electrostatic confining potential, which is experimentally used to define edges in two-dimensional (2D) electron gases buried in semiconductor heterostructures, enriches the picture with the concept of edge reconstruction (*8*). There, the Coulomb interaction energy dominates the confining potential, leading to a transformation of the edge states into a series of wide compressible channels separated by incompressible strips. In the opposite case of a sharp potential, the Coulomb interaction is not relevant, and the single-particle picture is valid. Edge reconstruction mechanisms have further proven to be of paramount importance in the fractional QH regime where additional co- and/or counterpropagative or even neutral modes (*9*–*11*) can emerge and complexify charge and heat transport (*12*–*14*).

Nonreconstructed edge states can substantially clarify QH edge transport with virtually ideal 1D edge states (*15*) and new regimes of intra- and interchannel interactions. Contrary to semiconductor heterostructures, 2D crystalline materials like graphene, for which physical edges are crystal edges, may be archetypical systems hosting such edge states. For graphene and its massless, linear band structure, QH edge states without confining electrostatic potential are expected to be the exact eigenstates of the Dirac equations derived with vanishing boundary conditions at the armchair or zigzag edge (*16*–*18*). Akin to Halperin’s original prediction (*2*), these solutions for edges states are maximally confined to a few magnetic lengths $l_{B}=\sqrt{\hbar /eB}$ (ℏ is the reduced Planck constant, *e* is the electron charge, and ***B ***is the magnetic field) from the crystal edge, leaving no room for edge reconstruction.

Here, we unveil the real-space structure of the QH edge states of graphene lying on an insulating hexagonal boron nitride (hBN) flake and evidence the absence of edge reconstruction by performing scanning tunneling spectroscopy up to the graphene crystal edge under strong perpendicular magnetic field. We achieved this by overcoming the long-standing experimental challenge (*19*–*29*) of approaching a scanning tunneling tip to the edge without crashing it on the insulating substrate that borders the graphene flake by means of a prior localization of the graphene edge by atomic force microscopy (AFM). We purposely used a homemade hybrid scanning microscope (*30*) capable of operating alternatively in AFM and scanning tunneling microscopy (STM) mode, thanks to a PtIr STM tip glued onto a piezoelectric tuning fork acting as a force sensor (*31*, *32*) for AFM (see Fig. 1A). Our sample schematized in Fig. 1 (A and B) consists of a graphene monolayer deposited on an hBN flake sitting on a Si/SiO_2_ substrate that serves as a back-gate electrode (see Methods). The graphene flake is contacted by a Cr/Pt/Au trilayer that allows to apply a voltage bias *V*_b_ and collect a tunnel current *I*_t_ via the STM tip. All experiments presented here are performed at a temperature of 4.2 K and a perpendicular magnetic field of 14 T.

## RESULTS

### QH edge states spectroscopy

Figure 1E displays an STM topographic image taken in constant current mode of the graphene edge, initially coarsely located by AFM (see fig. S1). The height profile of this image (Fig. 1D) shows a large flat area and a slight bump on the left part of the scan. This bump results from the tip-graphene interaction lifting up the graphene edge when the tip is right above it (*33*). This bump allows us to locate the edge of the graphene crystal with an accuracy of a few nanometers (see the Supplementary Materials). To the left of the bump, the tip dips toward the hBN substrate, on which a tip crash is avoided by a height limit of the STM controller. Atomic-scale imaging of the honeycomb lattice shown in Fig. 1F gives insight into the graphene lattice termination. The edge orientation in Fig. 1E, which is reported in Fig. 1F with the blue line, indicates an armchair termination.

**Fig. 2.**
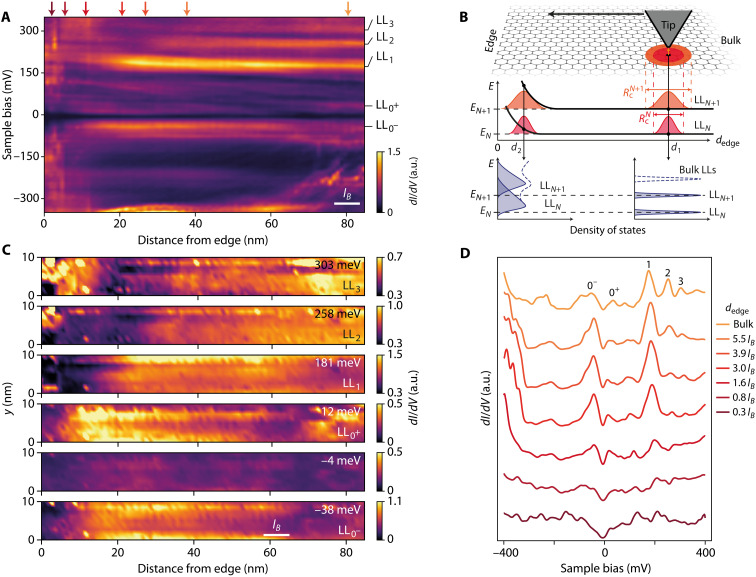
Sharp QH edge states. (**A**) Evolution of the tunneling conductance *dI*_t_/*dV*_b_ as a function of the distance from graphene edge measured at charge neutrality (*V*_g_ = −5.4 V). The half-filled zeroth Landau level is split into two sublevels LL_0^+^_ and LL_0^−^_ due to QH ferromagnetism (*30*). (**B**) Schematics of the tunneling into QH edge states. Because of the spatial extent $R_{c}^{N}=l_{B}{\left(2|N|+1\right)}^{1/2}$ of the LL*_N_* wave functions, the tunneling electrons probe at one point contributions from all states up to distances of about $R_{c}^{N}$ (red and orange Gaussians in the middle for LL*_N_* and LL_*N*+1_, respectively). The resulting density of states features sharp Landau level peaks in the bulk, i.e., at distance *d*_1_ from the edge, and a smooth profile close to the edge, at a distance *d*_2_ ∼ *l_B_*, due to the energy broadening of Landau levels (*16*). In addition, when approaching the edge, the tip starts to probe the edge states of the lower Landau levels, pushed at higher energies by the presence of the physical edge, and overlapping with the highly degenerate bulk states. The resulting peaks in the density of states thus exhibit a spectral weight redistribution toward higher energies, which leads to a suppression of the Landau level peak height in the tunneling conductance (bottom; in solid blue, each individual *N* and *N* + 1 Landau level peak; and in dashed blue, the overall density of states). (**C**) Spatial maps of the tunneling conductance *dI*_t_/*dV*_b_ at the energies of the Landau levels. (**D**) Individual spectra taken from (A) at different distances from the edge indicated by the color-coded arrows in (A). a.u., arbitrary units.

The central result of this work is shown in Fig. 2, which presents the evolution of the Landau levels upon approaching the immediate proximity of the graphene edge in the region shown in Fig. 1E, under a magnetic field of 14 T. We first study charge-neutral graphene by tuning the density with the back-gate voltage set at *V*_g_ = −5.4 V. Tunneling spectroscopy of Landau levels (*34*–*37*) results in a series of peaks in the tunneling conductance *G*(*V*_b_) = *dI*_t_/*dV*_b_ that is proportional to the local density of states. We show in Fig. 2A the tunneling conductance *G*(*d*_edge_, *V*_b_) as a function of tip distance perpendicular to the graphene edge *d*_edge_ and bias voltage *V*_b_. Far from the edge, Landau levels are readily identified as bright conductance peaks that we label LL*_N_*, where *N* is the Landau level index. These conductance peaks are conspicuously stable upon approaching the edge on the left of the figure. Within 40 nm from the edge, we observe a suppression of the Landau level peak heights (see individual spectra in Fig. 2D) starting at distances that depend on the Landau level (the higher the Landau index, the further from the edge). Figure 2C shows spatial maps of the tunneling conductance at the voltage bias of the Landau level peaks. For each Landau level peak, darker areas corresponding to Landau level peak suppression appear further and further from the edge as the Landau level index increases.

These findings contrast with the expectation for a smooth confining potential at the edges, for which the Landau level spectrum would have continuously shifted in energy, following the confining potential as the edge is approached. Because the tunneling conductance probes states on the scale of the electron wave function, that is, the cyclotron radius $R_{c}^{N}=l_{B}{\left(2|N|+1\right)}^{1/2}$ for Landau level index *N*, the suppression of the Landau level peaks, here, reflects a spreading of the spectral weight to higher energy due to an abrupt edge state dispersion at the physical edge, on a very short scale of the order of the magnetic length (see Fig. 2B). This suppression of the tunneling density of states of the Landau levels, which has been observed on graphene on a conductive graphite substrate (*27*), is therefore direct evidence of QH edge states sharply confined at the edges. Ultimately, on the last few nanometers from the edge, the Landau level peaks disappear completely, and the redistribution of Landau level spectral weight yields a V shape–like tunneling density of states (see Fig. 2D).

In this measurement, we have set the Fermi level at charge neutrality, that is, at Landau level filling factor ν = 0, which leads to a splitting of the zeroth Landau level (see split peaks labeled LL_0^+^_ and LL_0^−^_ in Fig. 2A) with the opening of an interaction-induced gap at *V*_b_ = 0 V [see (*30*)]. This splitting signals the broken-symmetry state (*38*) at charge neutrality with the Kekulé bond order (*30*, *39*, *40*). We identified the Kekulé bond order at 20 nm of the edge in Fig. 1G, indicating that this broken-symmetry state, which develops in the bulk, is robust even in the very proximity of the edge (*41*).

**Fig. 3.**
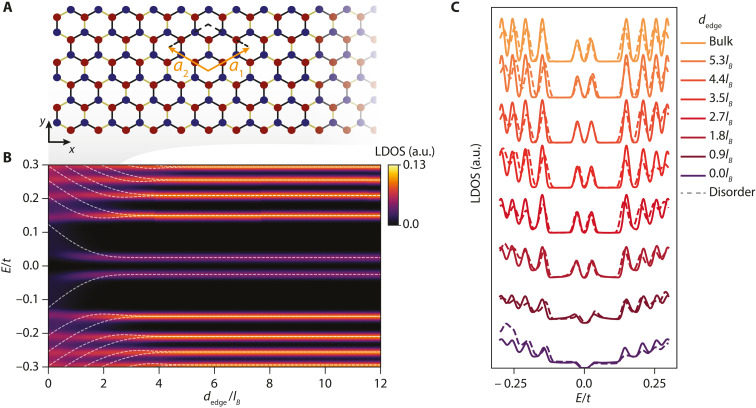
Theoretical tunneling density of states. (**A**) Schematic of the simulated edge geometry. We considered a Kekulé bond order (*30*, *39*, *40*) as broken-symmetry state (*38*), with lattice vectors that triple the unit cell compared to pristine graphene. (**B**) Corresponding local density of states (LDOS) as a function of the distance from the armchair graphene edge, *d*_edge_, normalized by *l_B_*, for charge-neutral graphene. The Kekulé bond order splits the zeroth Landau level into two sublevels LL_0^+^_ and LL_0^−^_ with an energy gap chosen to match the experimentally measured value of 50 meV (*30*). The white dashed lines are the numerically computed Landau levels of graphene nanoribbon with armchair termination. Because of position momentum locking, the Landau Levels disperse as they approach the physical edge, as sketched in Fig. 1B. (**C**) Individual spectra taken from (B) at different distances from the edge. Solid lines show cuts at different *d*_edge_, while dashed lines show spectral asymmetry emerging from a single disorder realization of an on-site disorder potential with strength *W*/*t* = 0.3 (see Methods).

To substantiate our finding, we performed numerical simulations of the local density of states of a charge-neutral graphene ribbon with an armchair edge under perpendicular magnetic field (see Fig. 3A) (*16*–*18*). We computed the Landau levels of the lattice Hamiltonian of nearest-neighbor hopping energy *t*. We assumed a Kekulé bond order with a gap at half-filling of the zeroth Landau level of 50 meV, as measured experimentally (*30*). The eigenstates for a ribbon with periodic boundary conditions along $\hat{y}$ are shown in Fig. 3B as white dashed lines. The Landau level eigenstates disperse as their average *x* position, locked to their *k_y_* momentum, approaches the physical edge of graphene (*42*), as schematized in Fig. 1B. Figure 3B shows the clean local density of states, which integrates the eigenstates weighted by the amplitude of the wave functions, as a function of the distance to the edge normalized by *l_B_*, *d*_edge_/*l_B_*, averaged over each unit cell (see Methods). The range of *d*_edge_/*l_B_* coincides with the range of displacement in Fig. 2A, allowing direct comparison with the experimental data. The resulting Landau level peaks are suppressed at higher values of *d*_edge_ the higher their Landau level index, and on the same spatial scale as observed experimentally in Fig. 2A. This reduction of spectral weight is more visible in Fig. 3C where we plot spectra for different *d*_edge_ (solid lines) including a single realization of on-site disorder (dashed lines). The latter breaks the particle-hole symmetry of the spectrum and thus may contribute to the asymmetries observed in the zeroth Landau level peaks.

### On the charge accumulation on the edges

The question of charge carrier homogeneity is critical for graphene transport. A body of work has shown anomalous asymmetry in some transport properties supplemented by scanning probe investigations (*43*–*45*), which points to a charge carrier accumulation at the graphene edges. Its origin may be either electrostatic stray field of the back-gate electrode (*46*) or chemical doping due to edge treatments (etching) or dangling bonds. In the QH effect, such an accumulation could open up additional counterpropagative edge channels and produce dissipation (*44*–*46*).

In tunneling experiments, a charge inhomogeneity on the edge would result in an energy shift of the Landau level spectrum as a whole due to a local change of the Landau level filling factor. Our measurements in Fig. 2 provides a first insight on this issue with a remarkable stability of the Landau level peaks in energy that indicates that a possible charge accumulation is not large enough to depin the chemical potential from the zeroth Landau level (*30*). In particular, it is lower than the value δ*n* = 6.8 × 10^11^ cm^−2^ required to fill the zeroth Landau level and reach ν = 2 at 14 T, which would produce a visible energy shift of the Landau level spectrum that we do not observe.

**Fig. 4.**
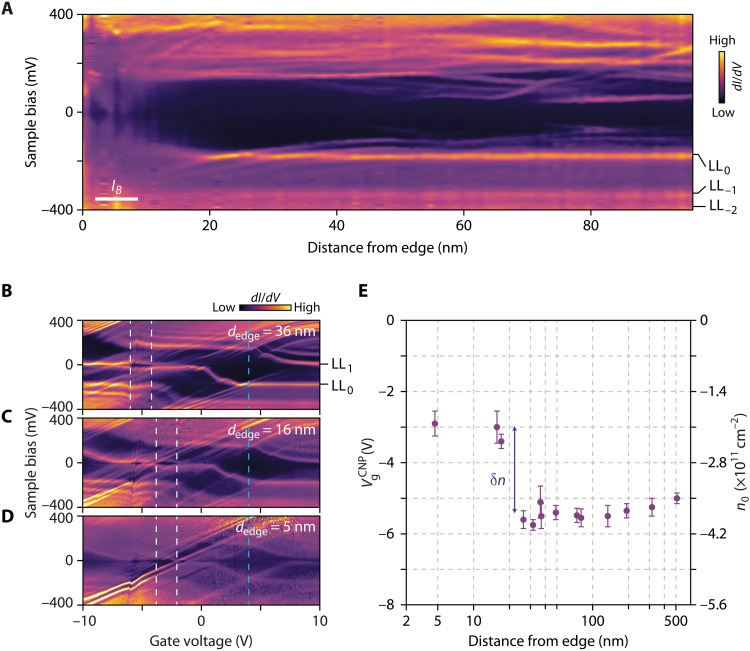
Charge density inhomogeneity on pristine edges. (**A**) Spatial evolution of the tunneling conductance up to the edge at filling factor ν = 2 for *V*_g_ = 4 V. (**B** to **D**) Tunneling conductance gate maps as a function of sample bias *V*_b_ and gate voltage *V*_g_. At a distance *d*_edge_ = 36 nm = 5.3 *l_B_* from the edge, in (B), we observe the staircase pattern of Landau levels of the graphene bulk. At closer distances from the edge, the Landau level peaks in the staircase pattern start to blur (C) and mostly vanish in (D). In the three panels, the opening of the ν = 0 gap as a function of *V*_g_ is indicated by white dashed lines, and the back-gate voltage of the charge-neutrality point $V_{g}^{\mathrm{CNP}}$ is identified by the maximum of the gap. The blue dashed lines indicate the spectra at *V*_g_ = 4 V, which coincide with the back-gate voltage of (A). The faint diagonal lines that translate into faint horizontal lines above the LL _0_ peak in (A) result from residual charging effects in the tunneling process. Their downward dispersion near the edge in (A) is consistent with the charge carrier variation in (E). (**E**) Evolution of $V_{g}^{\mathrm{CNP}}$ and charge carrier density *n*_0_ determined from tunneling conductance gate maps as a function of the distance from the edge *d*_edge_. The position of the charge carrier density shift coincides with the upward shift of the LL_0_ in (A). Error bars correspond to the range of gate voltage where the ν = 0 gap opens in the gate maps.

To enhance the sensitivity of the spectroscopy to possible charge inhomogeneities, we performed similar measurements at filling factor ν = 2 (*V*_g_ = 4 V), when the Fermi level is pinned by localized states in the cyclotron gap separating LL_0_ from LL_1_. There, because of the little density of localized states as compared to the highly degenerate Landau levels, a small variation of charge density would result in a substantial shift of the Landau levels in the tunneling spectra. Figure 4 displays the spatial evolution of the tunneling conductance up to the edge at ν = 2 and 14 T. As in Fig. 2, the Landau level peaks (LL_0_, LL_−1_, and LL_−2_) stay at the same energy over the scan and vanish at about 20 nm from the edge, clearly indicating the absence of charge accumulation. We further performed systematic gate-tuned tunneling spectroscopy maps at various locations, from 500 to 5 nm from the edge (see the Supplementary Materials). Figure 4 (B to D) displays three of these maps taken close to the edge. We observe in Fig. 4B the usual staircase pattern of the Landau level peaks due to the successive pinning of the Fermi energy in the Landau levels (*30*, *47*, *48*), which allows us to precisely identify the back-gate voltage of the charge neutrality point $V_{g}^{\mathrm{CNP}}$. As shown in Fig. 4E that displays $V_{g}^{\mathrm{CNP}}$ as a function of the distance from the edge, there is no charge accumulation from 500 to 20 nm to the edge, and only within 20 nm of the edge we measure a variation δ*n* = (−1.5 ± 1.1) × 10^11^ cm^−2^.

Such a charge density variation near the edge at 14 T yields a little variation δν = 0.4 of local filling factor, which would have no consequence on the QH edge transport properties. Extrapolating at lower field, however, δν = 2 would be reached at a magnetic field of 3 T, thus potentially affecting edge transport with additional modes. However, the very small spatial scale of this charge accumulation cannot explain recent scanning probes experiments evidencing indirect, sometimes out-of-equilibrium responses within hundreds of nanometers from the edge (*44*, *45*). We conjecture that this charge accumulation in our particular case is related to the tip-graphene interaction when the tip reaches and lift up the graphene edge (see section SIII).

## DISCUSSION

The issue of charge accumulation on the edge and the ensuing emergence of upstream modes (*44*, *45*) were put forth as an alternative interpretation (*49*) for the signature of helical edge transport in charge-neutral graphene (*50*, *51*). Although we cannot exclude that the stray field of the back-gate electrode may accumulate charges at high back-gate voltages, that is, away from charge neutrality point, and over a long distance (*46*), our results show that this accumulation is absent at low back-gate voltage, thus invalidating the doubts raised (*49*) on the existence of the QH topological insulator phase in charge-neutral graphene (*50*, *51*). Still, it may be interesting to revisit nonlocal transport in nonlinear regime (*49*) in view of the exact spatial structure of the QH edge states in graphene.

Regarding edge reconstruction, a wealth of fractional and integer QH states exhibits complex sequences of reconstructed edge channels, including additional integer and/or fractional as well as neutral modes (*9*–*11*). Whereas the smooth electrostatic potential in GaAs and other semiconductors reconstructs edge states into wide compressible stripes of the order of ⁓100 nm [see (*28*)], the graphene QH edge states confined on a very short length scale, at few magnetic lengths on the physical edge, pose new constraints and limits for such a reconstruction, opening the investigation of universal transport and thermal properties (*15*). Moreover, in such a strongly confined configuration, an enhancement of inter-edge states interactions can be expected, which makes the picture of independent chiral channels irrelevant in this case, thus affecting charge and heat equilibration (*52*–*54*). This should affect QH interferometry (*55*) in graphene systems (*56*, *57*) and other coherent experiments (*5*), for which the independence, exact positions, and nature of edge modes are crucial parameters to address anyon physics and other interaction-driven phenomena, such as charging effects (*58*), spin-charge separation (*59*), or electron pairing (*60*). Note: A very recent work (*61*) reports a complementary tunneling spectroscopy study of electrostatically defined QH edge states at a pn junction.

## METHODS

### Sample fabrication

The graphene/hBN heterostructure was assembled from exfoliated flakes with the van der Waals pickup technique using a polypropylene carbonate polymer (*62*). The stack with graphene on top of the hBN flake was deposited using the method described in (*63*) on a highly p-doped Si substrate with a 285-nm-thick SiO_2_ layer. Electron beam lithography using a poly(methyl methacrylate) resist was used to pattern a guiding markerfield on the whole 5 mm–by–5 mm substrate to drive the STM tip toward the device and to locate the graphene edge. Cr/Pt/Au electrodes contacting the graphene flake were also patterned by electron beam lithography and metalized by e-gun evaporation. The sample was thermally annealed at 350°C in vacuum under a halogen lamp to remove resist residues and clean graphene before being mounted into the STM where it was heated in situ during the cooling to 4.2 K.

### Measurements

Experiments were performed with a homemade hybrid STM and AFM operating at a temperature of 4.2 K in magnetic fields up to 14 T. The sensor consists of a hand-cut PtIr tip glued on the free prong of a tuning fork, the other prong being glued on a Macor substrate. Once mounted inside the STM, the tip is roughly aligned over the sample at room temperature. The AFM mode was used first for coarse navigation at 4.2 K on the sample surface to align the tip onto graphene and then for locating coarsely the graphene edge; see the Supplementary Materials. The STM imaging in constant-height mode of the edge, done subsequently, yields a fine identification. Scanning tunneling spectroscopy was performed using a lock-in amplifier technique with a modulation frequency of 263 Hz and root mean square modulation voltage between 1 and 5 mV depending on the spectral range of interest. Current imaging tunneling spectroscopy (CITS) measurements were acquired by starting far from the edge, with a grid whose slow *x* axis is perpendicular to the edge direction (as imaged by STM) and the *y* axis is parallel to the edge with a size of a few tens of nanometers. A safety condition is added to the tip vertical *z*-position controller to prevent the crashing into the hBN flake beyond the graphene edge: if the *z* position reaches a threshold (typically 3 nm below the *z* position of the tip estimated close to the edge), the tip is withdrawn and the CITS ends. Imaging of the Kekulé bond order was carried out in STM constant-height mode after tuning the graphene to charge neutrality with the back gate, at a bias voltage corresponding to the energy of the LL_0_+ peak [see (*30*) for details].

### Theoretical simulations

To compute the local density of states shown in Fig. 3, we use the simulation software Kwant (*64*). First, we create a honeycomb lattice in a square system of size *L_x_* × *L_y_* = 130 × 130, in units of graphene’s lattice constant *a*. The unit cell for the Kekulé order is tripled compared to pristine graphene and is defined by the reciprocal vectors $a_{1}=a(3\sqrt{3}/2,3/2)$ and $a_{2}=a(-3\sqrt{3}/2,3/2)$; see Fig. 3A. To calculate the local density of states ρ(*E*, *x*) at a given energy *E* and Kekulé unit cell *x*, we average over the six sites weighted by the corresponding wave function, ρ(*E*, *x*) = ∑_α_ ‍ ∣ψ_α_(*x*)∣^2^δ(*E* − *E*_α_), where α runs over the six unit cell sites. We compute the local density of states spectra, shown in Fig. 3B as a color map, using the kernel polynomial method (*65*) with a target energy resolution of Δ*E*/*t* = 0.005 and a magnetic field of ϕ/ϕ_0_ = 0.005 in units of the magnetic flux ϕ_0_ = *h*/*e*. The dashed line spectra of Fig. 3B maps are obtained for a finite nanoribbon of width *L_y_*, with an armchair edge parallel to the $\hat{y}$ direction, as in (*42*). We allow the edge to be misaligned with the Kekulé lattice vectors, as observed experimentally in Fig. 1 (F and G). Last, the solid lines in Fig. 3C show cuts of the local density of states spectra shown in Fig. 3B. The dashed lines are calculated adding a single disorder realization obtained by adding a random on-site potential *V*_dis_ at each site to the clean local density of states spectra described above. The disorder strength at each site is drawn from a uniform distribution in the interval [−*W*, *W*] with *W*/*t* = 0.3.

## Supplementary Materials

### This PDF file includes:

Supplementary Text Figs. S1 to S7 References
