# Supplementary material for: Absence of edge reconstruction for quantum Hall edge channels in graphene devices
Source: Sci Adv. 2023 May 12;9(19):eadf7220. doi: 10.1126/sciadv.adf7220 (PMC10181179; doi:10.1126/sciadv.adf7220)
Supplement: Supplementary file 1 — Supplementary Text Figs. S1 to S7 References [file sciadv.adf7220_sm.pdf]

Science Advances
AAAS

Supplementary Materials for

Absence of edge reconstruction for quantum Hall edge channels in graphene devices

Alexis Coissard *et al.*

Corresponding author: Benjamin Sacépé, benjamin.sacepe@neel.cnrs.fr

Sci. Adv. **9**, eadf7220 (2023)
DOI: 10.1126/sciadv.adf7220

This PDF file includes:

Supplementary Text
Figs. S1 to S7
References

I. SAMPLE DETAILS AND AFM MAPPING

The sample AC04 studied is this work is a heterostructure made of a graphene sheet atop a hexagonal boron nitride (hBN) flake, assembled by van der Waals stacking, and then deposited on a $p^{++}Si/SiO_2$ substrate to enable back gating of the charge carrier density in graphene. The voltage bias V_b is applied using a Cr/Pt/Au contact patterned by e-beam lithography and covering partially the graphene sheet, leaving a large fraction of the perimeter accessible by the tip for imaging and tunneling spectroscopy of the edge states, see Fig. S1A and B. The graphene bulk properties of this sample have been presented in Ref. [30].

The STM tip is brought atop the graphene sheet by AFM imaging of the coding markerfield patterned on the whole chip surface. This guiding process is done after about ten AFM images. An AFM mapping of graphene and its boundary with the underlying hBN performed at $B = 14$ T is shown in Fig. S1C. High-resolution AFM images of some edges are placed in overlay. These images reveal that the vacuum annealing employed to clean the graphene left some resist residues

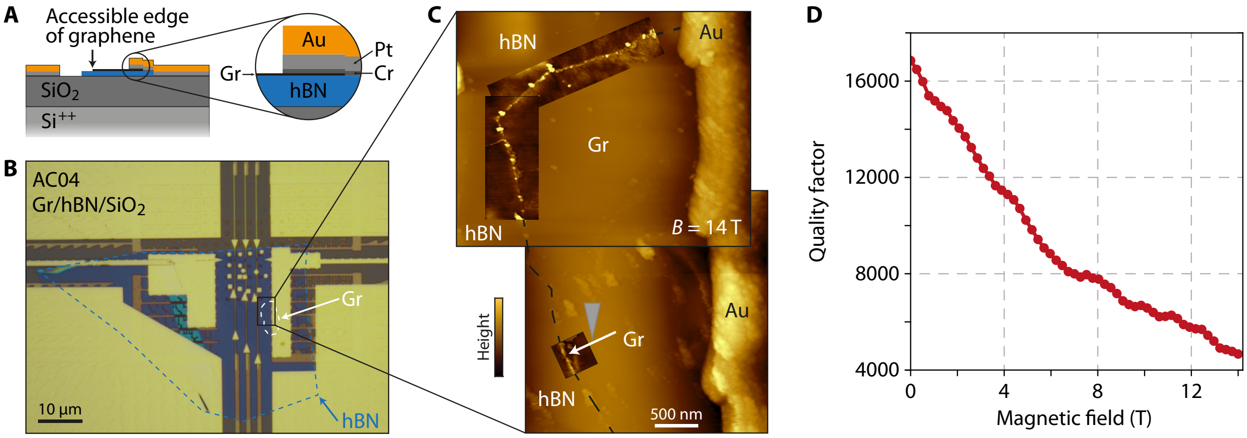

Fig. S1: Sample AC04 and AFM mapping. **A**, The graphene/hBN heterostructure is deposited on a Si^{++}/SiO_2 substrate that serves as a back-gate electrode to tune the charge carrier density. Graphene is biased with a Cr/Pt/Au contact patterned by e-beam lithography on one of its edge, leaving others accessible for the tunneling spectroscopy of QH edge states. **B**, Optical image of the device. The graphene and hBN flakes are outlined by white and blue dashed lines, respectively. **C**, AFM mapping of the graphene sheet at $T = 4.2$ K and $B = 14$ T, with three high-resolution images of the edges. The gold contact used to bias graphene is visible on the right of the images. The edge studied in this work is indicated by the white arrow. **D**, Evolution of the quality factor Q of the tuning fork with magnetic field.

that have migrated toward the edges, forming bright spots in-between which edges are clean. In this work we focus on the edge indicated by the white arrow, which is also the direction of the Current Imaging Tunneling Spectroscopy (CITS) measurement grids performed from the bulk of graphene to the edge. Note that the tuning fork we used here still displays a relatively high quality factor in magnetic field, with $Q \sim 4000$ at 14 T (Fig. S1D).

II. LOCALIZATION OF GRAPHENE EDGES ON hBN

We show in Fig. S2A STM image at $B = 14$ T of the edge of graphene indicated by the white arrow in Fig. S1C, which provides a very accurate identification of the edge position. It is obtained in constant height mode: before STM imaging, we approach the STM tip in tunneling contact with the graphene in order to measure the setpoint tunneling current (typically 1 nA) and next switch off the Z-regulation for imaging. This mode allows a safe imaging of graphene edge since the tip would not crash down on the insulating hBN, but would rather simply measure zero tunneling current as seen on the left part of the STM image in Fig. S2. However, on the very edge of the graphene flake, the honeycomb lattice is not resolved due to the instability of the tunneling current at this location. As a result, the meaningful information in Fig. S2 is the vanishing of the tunneling current when the tip reaches the hBN, which constitutes a clear identification of the edge location with nanometer-scale precision. Using this image of the edge, we can estimate its direction as indicated by the dashed blue line in Fig. S2. We report this line on the honeycomb lattice of the inset taken a few nanometers away and identify the armchair orientation for this edge.

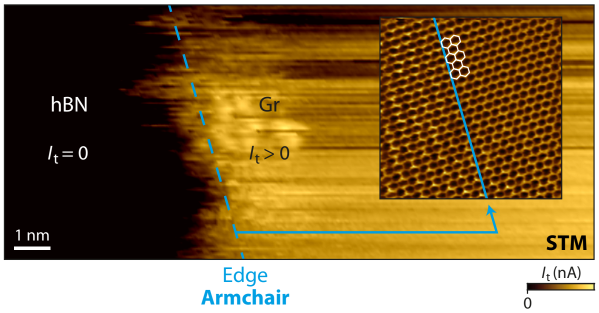

Fig. S2: Locating graphene armchair edge. $16 \times 7\,\mathrm{nm}^2$ STM image in constant height mode of a graphene edge. On the left side the tunneling current vanishes, indicating the tip is atop hBN. Inset : $5 \times 5\,\mathrm{nm}^2$ STM image of the honeycomb lattice a few nanometers away from the edge. When reporting the edge direction (blue dashed line) on the honeycomb lattice, it coincides with an armchair edge orientation.

We believe that the instability of the tunneling current measured on the very edge of the graphene in Fig. S2 stems from the local lifting of the graphene sheet edge from the hBN flake, each time the STM tip scans over it, due to electrostatic interactions with the tip.

III. TIP-INDUCED LIFTING OF THE GRAPHENE EDGE AND DEFINITION OF THE EDGE POSITION

We discuss here another way to locate the edge by means of a CITS grid spectroscopy measurement of the spatial dispersion of the Landau level (LL) spectrum toward the boundary. The grid spectroscopy is set to start far away in graphene bulk and to finish a few nanometers beyond the edge, previously located with STM images. Moreover, the slow x-axis direction of the grid is chosen to be perpendicular to the edge. A safety condition is added to the Z-controller to prevent the tip from crashing into hBN : if the Z-position of the tip goes below a threshold (typically 3 nm below the Z-position of the tip estimated close to the edge), the tip is withdrawn and the CITS ends.

We show in Fig. S3A the topographic map $z(d_{\text{edge}}, y)$ obtained from a CITS toward the graphene armchair edge identified in Fig. S2. d_{edge} is the distance from the armchair edge, while y is the lateral coordinate parallel to the edge. The topographic map features a clean and flat bulk graphene on a $80 \times 10\,\text{nm}^2$ area next to the edge. When the tip is situated a few nanometers away from the edge, the $z(d_{\text{edge}}, y)$ map reveals inhomogeneous bright spots. Though one can first think about residues, the small height of these spots, around $1 - 3$ Å, rules out this hypothesis.

We rather attribute these large spots to the lifting of the edge of the graphene sheet, as illustrated in Fig. S3D. The attractive van der Waals force of the tip was shown [33] to lift locally a graphene sheet lying on a SiO_2 substrate on a typical height of 1 Å. Although we do not observe such lifting in Fig. S3A in bulk graphene (either because the deformation follows the tip such that we eventually observe an overall flat background, or because the deformation of the graphene sheet on hBN is more difficult, since the adhesion interactions between both materials are more important than between graphene and SiO_2), we can assume that the graphene flake is more easily deformed at the edge by the force of the tip, and therefore the lifting is larger there than in the bulk.

The lifting of the edge is well visible in the height profile of Fig. S3B, obtained by averaging the topographic map along the y direction (parallel to the edge). The z profile features a flat region corresponding to bulk graphene (with variations of less than 1 Å), and a hump of 3 Å height at the edge. After that, the tip quickly moves down by several nanometers until it meets the

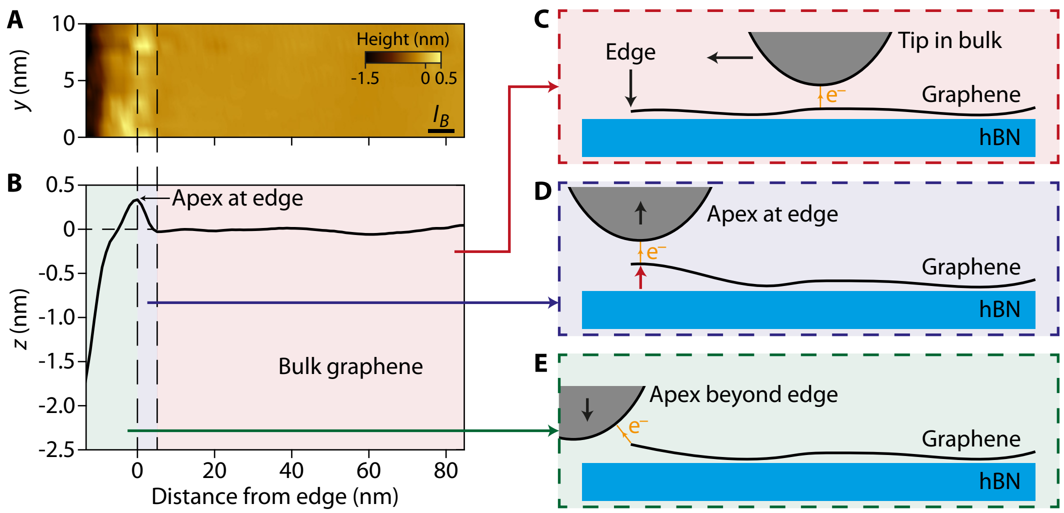

Fig. S3: CITS to the graphene edge: locating the edge. A, B, $z(d_{\mathrm{edge}}, y)$ topographic map (A) and $z(d_{\mathrm{edge}})$ profile (B) obtained from a CITS toward the armchair edge and beyond. We distinguish three regimes : the flat horizontal profile (red area) where the electrons tunnel in bulk graphene, see **C**, the sharp increase of z (blue region) where the electrons tunnel from the tip apex very close to the edge in a situation where the sheet is strongly lifted, see **D**, and the decrease of z when the tip apex is moved outside the graphene sheet, with a residual tunneling between the edge of graphene and other atoms on the side of the tip, see **E**, preventing the tip from crashing into hBN. The edge position is taken at the position of the maximum of the topographic profile.

safety condition of the Z-controller, which stops the CITS. We attribute this lowering of the z position to the fact that the tip apex has gone beyond the edge of graphene, but tunneling remains possible with some other higher atoms of the tip close to the apex, see Fig. S3D. This makes the measurement of a tunneling current possible even when the apex itself is lying on hBN, yet this current is highly unstable.

From this model we assume the position of the edge of graphene (*i.e.* the tip apex is atop the edge) is given by the maximum of the hump in the z profile, and from this origin we compute d_{edge} the distance from the edge, which we use in the main text and the following figures.

IV. ADDITIONAL TUNNELING CONDUCTANCE MAPS AT THE EDGE

We show in this section two additional tunneling conductance maps acquired along the same armchair edge, but a few tens of nanometers away from the map shown in Fig. 2 of the main text. The back-gate voltage is fixed at $V_{\mathrm{g}} = -5$ V, corresponding to filling factor $\nu = 0$.

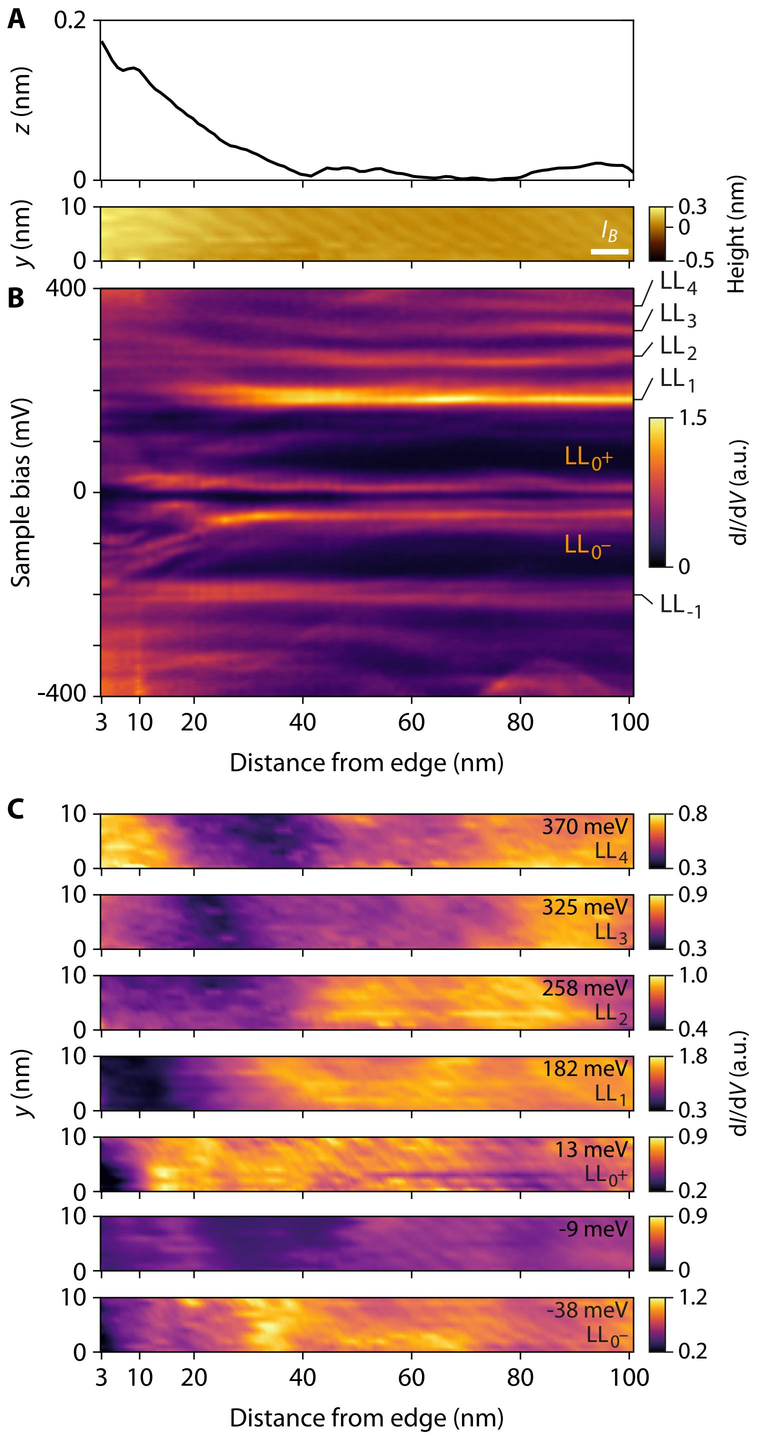

Fig. S4: Landau level spectroscopy toward armchair edge. A, Topographic profile $z(d_{\mathrm{edge}})$ and map $z(d_{\mathrm{edge}}, y)$ near the graphene edge ($l_B = 6.85$ nm at $B = 14$ T). **B**, Tunneling conductance as a function of the distance to the edge and sample bias, toward the edge. **C**, Tunneling conductance spatial maps at the different LL energies. The panel at $V_{\mathrm{b}} = -9$ mV shows the spatial map of the $\nu = 0$ gap.

In Figs. S4 and S5, the panels (a) show the topographic map $z(d_{\text{edge}}, y)$ and the profile $z(d_{\text{edge}})$ obtained by averaging the map on the lateral y-dimension. Bulk graphene appears flat and clean, with a corrugation of at most 1 Å on a distance of 100 and 300 nm, respectively. When approaching the edge on the left, $z(d_{\text{edge}})$ increases by around $2-3$ Å due to the tip-induced lifting of the graphene sheet edge. In Fig. S4 the CITS grid spectroscopy did not go beyond the edge: the edge position is rather roughly estimated using the STM image in Fig. 1D from the main text. The same goes for Fig. S5.

Panels (b) show the tunneling conductance toward the armchair edge as a function of the distance to the edge and the sample bias. The same qualitative observations as that of the main text can be made for the two edges: the Landau level peaks do not disperse when approaching the edges but vanish. The splitting of the LL_0 is well visible and the gap stays open down to the edge where it even gets more pronounced.

Panel (c) in Fig. S4 shows the tunneling conductance as a function of the distance to the edge and the y-direction parallel to the edge, at different bulk Landau level energies E_N.

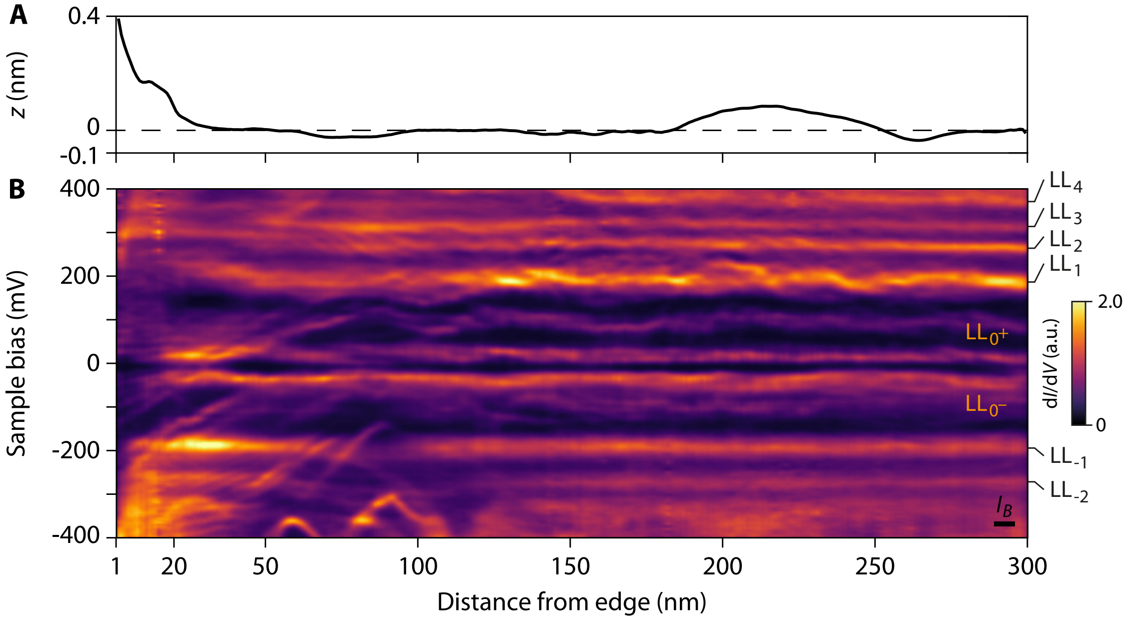

Fig. S5: Landau level spectroscopy toward armchair edge. A, Topographic profile $z(d_{\text{edge}})$ near the graphene edge ($l_B = 6.85$ nm at $B = 14$ T). **B**, Tunneling conductance as a function of the distance to the edge and voltage bias.

We now consider in more details the tunneling conductance map shown in Fig. S4B. We plot in Fig. S6A the evolution of the positions in energy E_N of the visible LL_N peaks and in Fig. S6B the variation of their height as a function of d_{edge}. The amplitude of the peaks decreases as we

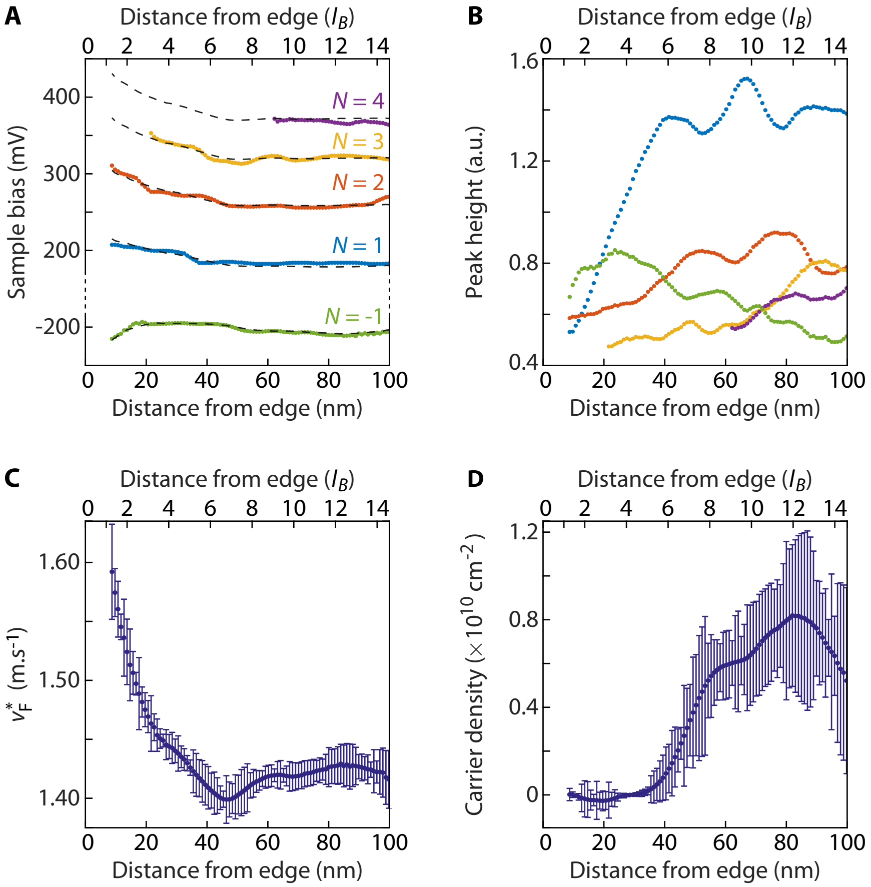

Fig. S6: Renormalized Fermi velocity. **A**, Peak energy for LL_N as a function of the distance from the edge, extracted from the LDOS map in Fig. S4. The dashed lines show the fitted energies using the parameter v_F^* obtained in (C). **B**, Peak height for the same LL_N (same color code). **C**, Evolution of the effective Fermi velocity v_F^* toward the edge obtained from the fit of LL_{-1}, LL_1, LL_2, LL_3 and LL_4 positions (when visible). **D**, Carrier density n computed from the Dirac point position E_D obtained from the same fit. n presents a residual bulk value of $n_0 \approx 7 \times 10^9\,\mathrm{cm}^{-2}$ and vanishes from 40 nm from the edge.

approach the edge until peaks merge into a V-shape background at the edge where they are no longer visible. In particular, LL_4 vanishes at $9\,l_B$ from the edge, LL_3 at $3\,l_B$ whereas LL_2 and $\mathrm{LL}_{\pm1}$ disappear at l_B. The amplitude of LL_1 also vanishes way faster than the other LL_N of higher index N. In addition to the peak vanishing at the edge, we can also notice in Fig. S4B and S6A a weak dispersion toward higher energy of the LL_N peaks close to the edge (on a length of around $\sim 6\,l_B$ from the edge), see Ref. [27].

Furthermore, we can fit the positions E_N of $\mathrm{LL}_{N\neq0}$ at each d_edge (for every visible LL at this point) with respect to equation $E_N = E_\mathrm{D} + \mathrm{sign}(N) v_\mathrm{F}^* \sqrt{2\hbar e|N|B}$ to extract an effective Fermi

velocity v_F^* and an estimate of the Dirac point position E_D as a function of d_{edge}. These results are shown in Figs. S6C for v_F^* and S6D for E_D, which is converted into charge carrier density n using $n = -\text{sign}(E_D)\frac{1}{\pi}[E_D/\hbar v_F^*]^2$. The bulk value $v^*_{F,bulk} = 1.42 \times 10^6$ m.s^{-1} is consistent with a renormalization of the Fermi velocity due to the enhancement of electron-electron interactions at charge neutrality [47, 48, 66], as characterized in a previous work [30] for the same sample AC04. Below $7\,l_B$ the effective Fermi velocity starts to increase toward the armchair edge due to the dispersion of the LL peaks, reaching $v^*_{F,edge} = 1.6 \times 10^6$ m.s^{-1} at l_B from the edge. As for the carrier density, we obtain a residual value $n_0 \approx 7 \times 10^9$ cm^{-2} in bulk graphene (in agreement with a back-gate voltage tuned at $\nu = 0$). Below 60 nm the density is seen to decrease and eventually vanishes at 40 nm $= 5\,l_B$ from the edge. A similar decrease of the density with respect to its bulk value has also been observed around l_B from graphene edge on graphite [27]. Finally, we use the $v_F^*(d_{edge})$ and $E_D(d_{edge})$ parameters to plot in Fig. S6A the fitted energies of each LL (black dashed lines). We notice a good agreement with the experimental points, especially for the dispersing parts.

V. TUNNELING CONDUCTANCE GATE MAPS AT DIFFERENT DISTANCES FROM THE EDGE

We show in this section additional tunneling conductance gate maps (Fig. S7) used to plot the evolution of the charge-neutrality point V_g^{CNP} as a function of the distance from the edge in Fig. 3C of the main text. V_g^{CNP} is estimated at the middle of the $\nu = 0$ gap opening when LL_0 pins the Fermi level at zero bias. This gap due to exchange interaction is indeed expected to be maximal at charge neutrality (i.e. half filling).

In each panel we indicate the opening of the $\nu = 0$ gap by yellow dashed lines, observed as :

- either as a typical gap opening between both $LL_{0\pm}$ such as in panel (i,j),

- either as a kink toward negative energies in the LL_{0-} peak around zero sample bias, when the LL_{0+} is not visible, such as in panels (b-h),

- either as a kink in the other LL peaks or charging peaks when not easily visible for LL_0, such as in panel (a).

Note that the opening of the $\nu = 0$ gap at zero sample bias also induces a shift in energy of other LL peaks (and also of charging peaks), which enables unambiguous identification of the charge-neutrality point. Still these shifts in energy do not occur strictly at constant gate voltage due to

tip-induced gating, see for instance the red dashed line in Fig. S7C.

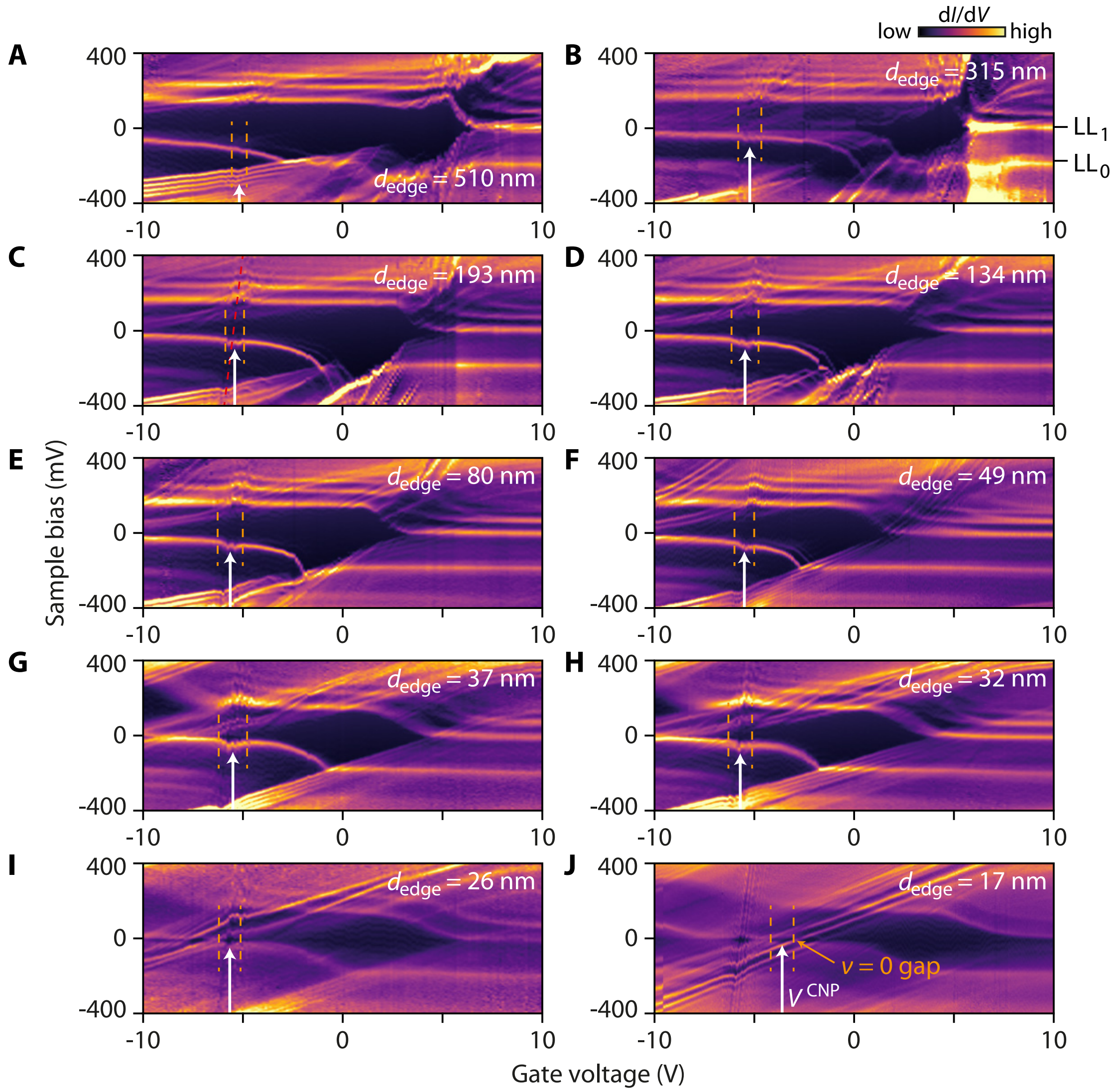

Fig. S7: Tunneling conductance gate maps at decreasing distances from the edge. For every panel, we identify the $\nu = 0$ gap that opens in charge-neutral graphene as a kink in the LL_0 peak when it pins the Fermi level at zero sample bias. The range of gate voltage where the gap opens is indicated by yellow dashed lines. The charge-neutrality point V_g^{CNP} is then assumed to be in the middle of this range, where the $\nu = 0$ gap is maximal, see white arrow. In panels (a-h), only the LL_{0-} peak is well visible, the LL_{0+} peak is hindered. In panel (A), the kink at charge-neutrality is hardly visible for LL_0, we rather identify it in the charging peaks below it.

REFERENCES AND NOTES

1. K. Von Klitzing, G. Dorda, M. Pepper, New method for high-accuracy determination of the fine-structure constant based on quantized Hall resistance. *Phys. Rev. Lett.* **45**, 494–497 (1980).

2. B. I. Halperin, Quantized Hall conductance, current-carrying edge states, and the existence of extended states in a two-dimensional disordered potential. *Phys. Rev. B* **25**, 2185–2190 (1982).

3. M. Büttiker, Absence of backscattering in the quantum Hall effect in multiprobe conductors. *Phys. Rev. B* **38**, 9375–9389 (1988).

4. C. Beenakker, H. van Houten, Quantum transport in semiconductor nanostructures. *Solid State Phys.* **44**, 1–228 (1991).

5. C. Bäuerle, D. C. Glattli, T. Meunier, F. Portier, P. Roche, P. Roulleau, S. Takada, X. Waintal, Coherent control of single electrons: A review of current progress. *Rep. Prog. Phys.* **81**, 056503 (2018).

6. H. Bartolomei, M. Kumar, R. Bisognin, A. Marguerite, J. M. Berroir, E. Bocquillon, B. Plaçais, A. Cavanna, Q. Dong, U. Gennser, Y. Jin, G. Fève, Fractional statistics in anyon collisions. *Science* **368**, 173–177 (2020).

7. J. Nakamura, S. Liang, G. C. Gardner, M. J. Manfra, Direct observation of anyonic braiding statistics. *Nat. Phys.* **16**, 931–936 (2020).

8. D. B. Chklovskii, B. I. Shklovskii, L. I. Glazman, Electrostatics of edge channels. *Phys. Rev. B* **46**, 4026–4034 (1992).

9. C. D. C. Chamon, X. G. Wen, Sharp and smooth boundaries of quantum Hall liquids. *Phys. Rev. B* **49**, 8227–8241 (1994).

10. C. L. Kane, M. P. A. Fisher, J. Polchinski, Randomness at the edge: Theory of quantum Hall transport at filling ν=2/3. *Phys. Rev. Lett.* **72**, 4129–4132 (1994).

11. U. Khanna, M. Goldstein, Y. Gefen, Fractional edge reconstruction in integer quantum Hall phases. *Phys. Rev. B* **103**, L121302 (2021).

12. V. Venkatachalam, S. Hart, L. Pfeiffer, K. West, A. Yacoby, Local thermometry of neutral modes on the quantum Hall edge. *Nat. Phys.* **8**, 676–681 (2012).

13. M. Goldstein, Y. Gefen, Suppression of Interference in quantum Hall Mach-Zehnder geometry by upstream neutral modes. *Phys. Rev. Lett.* **117**, 276804 (2016).

14. R. Bhattacharyya, M. Banerjee, M. Heiblum, D. Mahalu, V. Umansky, Melting of interference in the fractional quantum Hall effect: Appearance of neutral modes. *Phys. Rev. Lett.* **122**, 246801 (2019).

15. Z.-X. Hu, R. N. Bhatt, X. Wan, K. Yang, Realizing universal edge properties in graphene fractional quantum Hall liquids. *Phys. Rev. Lett.* **107**, 236806 (2011).

16. D. A. Abanin, P. A. Lee, L. S. Levitov, Spin-filtered edge states and quantum Hall effect in graphene. *Phys. Rev. Lett.* **96**, 176803 (2006).

17. L. Brey, H. A. Fertig, Edge states and the quantized Hall effect in graphene. *Phys. Rev. B* **73**, 195408 (2006).

18. D. A. Abanin, P. A. Lee, L. S. Levitov, Charge and spin transport at the quantum Hall edge of graphene. *Solid State Commun.* **143**, 77–85 (2007).

19. K. L. McCormick, M. T. Woodside, M. Huang, M. Wu, P. L. McEuen, C. Duruoz, J. S. Harris, Scanned potential microscopy of edge and bulk currents in the quantum Hall regime. *Phys. Rev. B* **59**, 4654–4657 (1999).

20. A. Yacoby, H. F. Hess, T. A. Fulton, L. N. Pfeiffer, K. W. West, Electrical imaging of the quantum Hall state. *Solid State Commun.* **111**, 1–13 (1999).

21. J. Weis, K. von Klitzing, Metrology and microscopic picture of the integer quantum Hall effect. *Phil. Trans. R. Soc. A* **369**, 3954–3974 (2011).

22. H. Ito, K. Furuya, Y. Shibata, S. Kashiwaya, M. Yamaguchi, T. Akazaki, H. Tamura, Y. Ootuka, S. Nomura, Near-field optical mapping of quantum Hall edge states. *Phys. Rev. Lett.* **107**, 256803 (2011).

23. K. Lai, W. Kundhikanjana, M. A. Kelly, Z.-X. Shen, J. Shabani, M. Shayegan, Imaging of coulomb-driven quantum Hall edge states. *Phys. Rev. Lett.* **107**, 176809 (2011).

24. M. E. Suddards, A. Baumgartner, M. Henini, C. J. Mellor, Scanning capacitance imaging of compressible and incompressible quantum Hall effect edge strips. *New J. Phys.* **14**, 083015 (2012).

25. P. Weitz, E. Ahlswede, J. Weis, K. von Klitzing, K. Eberl, Hall-potential investigations under quantum Hall conditions using scanning force microscopy. *Phys. E* **6**, 247–250 (2000).

26. G. Nazin, Y. Zhang, L. Zhang, E. Sutter, P. Sutter, Visualization of charge transport through Landau levels in graphene. *Nat. Phys.* **6**, 870–874 (2010).

27. G. Li, A. Luican-Mayer, D. Abanin, L. S. Levitov, E. Y. Andrei, Evolution of Landau levels into edge states in graphene. *Nat. Commun.* **4**, 1–7 (2013).

28. N. Pascher, C. Rössler, T. Ihn, K. Ensslin, C. Reichl, W. Wegscheider, Imaging the conductance of integer and fractional quantum Hall edge states. *Phys. Rev. X* **4**, 011014 (2014).

29. S. Kim, J. Schwenk, D. Walkup, Y. Zeng, F. Ghahari, S. T. Le, M. R. Slot, J. Berwanger, S. R. Blankenship, K. Watanabe, T. Taniguchi, F. J. Giessibl, N. B. Zhitenev, C. R. Dean, J. A. Stroscio, Edge channels of broken-symmetry quantum Hall states in graphene visualized by atomic force microscopy. *Nat. Commun.* **12**, 2852 (2021).

30. A. Coissard, D. Wander, H. Vignaud, A. G. Grushin, C. Repellin, K. Watanabe, T. Taniguchi, F. Gay, C. B. Winkelmann, H. Courtois, H. Sellier, B. Sacépé, Imaging tunable quantum Hall broken-symmetry orders in graphene. *Nature* **605**, 51–56 (2022).

31. F. J. Giessibl, S. Hembacher, M. Herz, C. Schiller, J. Mannhart, Stability considerations and implementation of cantilevers allowing dynamic force microscopy with optimal resolution: The qPlus sensor. *Nanotechnology* **15**, S79–S86 (2004).

32. J. Senzier, P. S. Luo, H. Courtois, Combined scanning force microscopy and scanning tunneling spectroscopy of an electronic nanocircuit at very low temperature. *Appl. Phys. Lett.* **90**, 043114 (2007).

33. A. Georgi, P. Nemes-Incze, R. Carrillo-Bastos, D. Faria, S. Viola Kusminskiy, D. Zhai, M. Schneider, D. Subramaniam, T. Mashoff, N. M. Freitag, A. Georgi, M. Liebmann, M. Pratzer, L. Wirtz, C. R. Woods, R. V. Gorbachev, Y. Cao, K. S. Novoselov, N. Sandler, M. Morgenstern, Tuning the pseudospin polarization of graphene by a pseudomagnetic field. *Nano Lett.* **17**, 2240–2245 (2017).

34. T. Matsui, H. Kambara, Y. Niimi, K. Tagami, M. Tsukada, H. Fukuyama, STS observations of Landau levels at graphite surfaces. *Phys. Rev. Lett.* **94**, 226403 (2005).

35. K. Hashimoto, K. Hashimoto, C. Sohrmann, J. Wiebe, T. Inaoka, F. Meier, Y. Hirayama, R. A. Römer, R. Wiesendanger, M. Morgenstern, Quantum Hall transition in real space: From localized to extended states. *Phys. Rev. Lett.* **101**, 256802 (2008).

36. Y. J. Song, A. F. Otte, Y. Kuk, Y. Hu, D. B. Torrance, P. N. First, W. A. de Heer, H. Min, S. Adam, M. D. Stiles, A. H. MacDonald, J. A. Stroscio, High-resolution tunnelling spectroscopy of a graphene quartet. *Nature* **467**, 185–189 (2010).

37. E. Y. Andrei, G. Li, X. Du, Electronic properties of graphene: A perspective from scanning tunneling microscopy and magnetotransport. *Rep. Prog. Phys.* **75**, 056501 (2012).

38. M. O. Goerbig, From the integer to the fractional quantum Hall effect in graphene. arXiv: 2207.03322v1 (2022). https://doi.org/10.48550/arXiv.2207.03322.

39. S.-Y. Li, Y. Zhang, L.-J. Yin, L. He, Scanning tunneling microscope study of quantum Hall isospin ferromagnetic states in the zero Landau level in a graphene monolayer. *Phys. Rev. B* **100**, 085437 (2019).

40. X. Liu, G. Farahi, C.-L. Chiu, Z. Papic, K. Watanabe, T. Taniguchi, M. P. Zaletel, A. Yazdani, Visualizing broken symmetry and topological defects in a quantum Hall ferromagnet. *Science* **375**, 321–326 (2022).

41. A. Knothe, T. Jolicoeur, Edge structure of graphene monolayers in the $\nu = 0$ quantum Hall state. *Phys. Rev. B* **92**, 165110 (2015).

42. P. K. Pyatkovskiy, V. A. Miransky, Spectrum of edge states in the $\nu = 0$ quantum Hall phases in graphene. *Phys. Rev. B* **90**, 195407 (2014).

43. Y.-T. Cui, B. Wen, E. Y. Ma, G. Diankov, Z. Han, F. Amet, T. Taniguchi, K. Watanabe, D. Goldhaber-Gordon, C. R. Dean, Z.-X. Shen, Unconventional correlation between quantum Hall transport quantization and bulk state filling in gated graphene devices. *Phys. Rev. Lett.* **117**, 186601 (2016).

44. A. Marguerite, J. Birkbeck, A. Aharon-Steinberg, D. Halbertal, K. Bagani, I. Marcus, Y. Myasoedov, A. K. Geim, D. J. Perello, E. Zeldov, Imaging work and dissipation in the quantum Hall state in graphene. *Nature* **575**, 628–633 (2019).

45. N. Moreau, B. Brun, S. Somanchi, K. Watanabe, T. Taniguchi, C. Stampfer, B. Hackens, Upstream modes and antidots poison graphene quantum Hall effect. *Nat. Commun.* **12**, 1–7 (2021).

46. P. G. Silvestrov, K. B. Efetov, Charge accumulation at the boundaries of a graphene strip induced by a gate voltage: Electrostatic approach. *Phys. Rev. B* **77**, 155436 (2008).

47. A. Luican, G. Li, E. Y. Andrei, Quantized Landau level spectrum and its density dependence in graphene. *Phys. Rev. B* **83**, 041405(R) (2011).

48. J. Chae, S. Jung, A. F. Young, C. R. Dean, L. Wang, Y. Gao, K. Watanabe, T. Taniguchi, J. Hone, K. L. Shepard, P. Kim, N. B. Zhitenev, J. A. Stroscio, Renormalization of the graphene dispersion velocity determined from scanning tunneling spectroscopy. *Phys. Rev. Lett.* **109**, 116802 (2012).

49. A. Aharon-Steinberg, A. Marguerite, D. J. Perello, K. Bagani, T. Holder, Y. Myasoedov, L. S. Levitov, A. K. Geim, E. Zeldov, Long-range nontopological edge currents in charge-neutral graphene. *Nature* **593**, 528–534 (2021).

50. A. F. Young, J. D. Sanchez-Yamagishi, B. Hunt, S. H. Choi, K. Watanabe, T. Taniguchi, R. C. Ashoori, P. Jarillo-Herrero, Tunable symmetry breaking and helical edge transport in a graphene quantum spin Hall state. *Nature* **505**, 528–532 (2014).

51. L. Veyrat, C. Déprez, A. Coissard, X. Li, F. Gay, K. Watanabe, T. Taniguchi, Z. Han, B. A. Piot, S. Sellier, B. Sacépé, Helical quantum Hall phase in graphene on $SrTiO_3$. *Science* **367**, 781–786 (2020).

52. S. K. Srivastav, R. Kumar, C. Spånslätt, K. Watanabe, T. Taniguchi, A. D. Mirlin, Y. Gefen, A. Das, Vanishing thermal equilibration for hole-conjugate fractional quantum Hall states in graphene. *Phys. Rev. Lett.* **126**, 216803 (2021).

53. R. Kumar, S. K. Srivastav, C. Spånslätt, K. Watanabe, T. Taniguchi, A. D. Mirlin, Y. Gefen, A. Das, Observation of ballistic upstream modes at fractional quantum Hall edges of graphene. *Nat. Commun.* **13**, 213 (2022).

54. G. Le Breton, R. Delagrange, Y. Hong, M. Garg, K. Watanabe, T. Taniguchi, R. Ribeiro-Palau, P. Roulleau, P. Roche, F. D. Parmentier, Heat equilibration of integer and fractional quantum Hall edge modes in graphene. *Phys. Rev. Lett.* **129**, 116803 (2022).

55. D. E. Feldman, B. I. Halperin, Fractional charge and fractional statistics in the quantum Hall effects. *Rep. Prog. Phys.* **84**, 076501 (2021).

56. C. Déprez, L. Veyrat, H. Vignaud, G. Nayak, K. Watanabe, T. Taniguchi, F. Gay, H. Sellier, B. Sacépé, A tunable Fabry–Pérot quantum Hall interferometer in graphene. *Nat. Nanotechnol.* **16**, 555–562 (2021).

57. Y. Ronen, T. Werkmeister, D. Haie Najafabadi, A. T. Pierce, L. E. Anderson, Y. J. Shin, Y. H. Lee, J. Bobae, K. Watanabe, T. Taniguchi, A. Yacoby, P. Kim, Aharonov–Bohm effect in

graphene-based Fabry–Pérot quantum Hall interferometers. *Nat. Nanotechnol.* **16**, 563–569 (2021).

58. B. I. Halperin, A. Stern, I. Neder, B. Rosenow, Theory of the Fabry-Pérot quantum Hall interferometer. *Phys. Rev. B* **83**, 155440 (2011).

59. T. Fujisawa, Nonequilibrium charge dynamics of tomonaga-luttinger liquids in quantum hall edge channels. *Ann. Phys.* **534**, 2100354 (2022).

60. H. K. Choi, I. Sivan, A. Rosenblatt, M. Heiblum, V. Umansky, D. Mahalu, Robust electron pairing in the integer quantum Hall effect regime. *Nat. Commun.* **6**, 1–7 (2015).

61. T. Johnsen, C. Schattauer, S. Samaddar, A. Weston, M. Hamer, K. Watanabe, T. Taniguchi, R. Gorbachev, F. Libisch, L. Morgenstern, Mapping quantum Hall edge states in graphene by scanning tunneling microscopy. *Phys. Rev. B* **107**, 115426 (2023).

62. L. Wang, I. Meric, P. Y. Huang, Q. Gao, Y. Gao, H. Tran, T. Taniguchi, K. Watanabe, L. M. Campos, D. A. Muller, J. Guo, P. KimHone, J. Hone, K. L. Shepard, C. D. Dean, One-dimensional electrical contact to a two-dimensional material. *Science* **342**, 614–617 (2013).

63. Y. Choi, J. Kemmer, Y. Peng, A. Thomson, H. Arora, R. Polski, Y. Zhang, H. Ren, J. Alicea, G. Refael, F. von Oppen, K. Watanabe, T. Taniguchi, S. Nadj-Perge, Electronic correlations in twisted bilayer graphene near the magic angle. *Nat. Phys.* **15**, 1174–1180 (2019).

64. C. Groth, M. Wimmer, A. R. Akhmerov, X. Waintal, Kwant: A software package for quantum transport. *New J. Phys.* **16**, 063065 (2014).

65. A. Weisse, G. Wellein, A. Alvermann, H. Fehske, The kernel polynomial method. *Rev. Mod. Phys.* **78**, 275–306 (2006).

66. S. Das Sarma, E. H. Hwang, W.-K. Tse, Many-body interaction effects in doped and undoped graphene: Fermi liquid versus non-Fermi liquid. *Phys. Rev. B* **75**, 121406(R) (2007).
